# U.S. Federal Agency Interests and Key Considerations for New Approach Methodologies for Nanomaterials

**DOI:** 10.14573/altex.2105041

**Published:** 2021-12-03

**Authors:** Elijah J. Petersen, Patricia Ceger, David G. Allen, Jayme Coyle, Raymond Derk, Natalia Garcia Reyero, John Gordon, Nicole Kleinstreuer, Joanna Matheson, Danielle McShan, Bryant C. Nelson, Anil K. Patri, Penelope Rice, Liying Rojanasakul, Abhilash Sasidharan, Louis Scarano, Xiaoqing Chang

**Affiliations:** 1 U.S. Department of Commerce, National Institute of Standards and Technology, Gaithersburg, MD, USA; 2 Integrated Laboratory Systems LLC, Research Triangle Park, NC, USA; 3 National Institute for Occupational Safety and Health, Health Effects Laboratory Division, Morgantown, WV, USA; 4 Current affiliation: UES, Inc., Dayton, OH, USA; 5 U.S. Army Engineer Research and Development Center, Vicksburg, MS, USA; 6 U.S. Consumer Product Safety Commission, Bethesda, MD, USA; 7 National Institute of Environmental Health Sciences, National Toxicology Program Interagency Center for the Evaluation of Alternative Toxicological Methods, Research Triangle Park, NC, USA; 8 U.S. Environmental Protection Agency, Office of Pesticide Programs, Washington, DC, USA; 9 U.S. Food and Drug Administration, National Center for Toxicological Research, Jefferson, AR, USA; 10 U.S. Food and Drug Administration, Center for Food Safety and Applied Nutrition, College Park, MD, USA; 11 U.S. Environmental Protection Agency, Office of Pollution Prevention and Toxics, Washington, DC, USA

## Abstract

Engineered nanomaterials (ENMs) come in a wide array of shapes, sizes, surface coatings, and compositions, and often possess novel or enhanced properties compared to larger sized particles of the same elemental composition. To ensure the safe commercialization of products containing ENMs, it is important to thoroughly understand their potential risks. Given that ENMs can be created in an almost infinite number of variations, it is not feasible to conduct *in vivo* testing on each type of ENM. Instead, new approach methodologies (NAMs) such as *in vitro* or *in chemico* test methods may be needed, given their capacity for higher throughput testing, lower cost, and ability to provide information on toxicological mechanisms. However, the different behaviors of ENMs compared to dissolved chemicals may challenge safety testing of ENMs using NAMs. In this study, member agencies within the Interagency Coordinating Committee on the Validation of Alternative Methods were queried about what types of ENMs are of agency interest and whether there is agency-specific guidance for ENM toxicity testing. To support the ability of NAMs to provide robust results in ENM testing, two key issues in the usage of NAMs, namely dosimetry and interference/bias controls, are thoroughly discussed.

## 1 Introduction

Engineered nanomaterials (ENMs) are materials with a size range, in at least one dimension, from 1 nm up to 100 nm (ASTM E2456-06, 2006; ISO, 2019) or are engineered to exhibit properties or phenomena (chemical, physical, or biological) that are attributable to their dimension(s), even if those dimensions fall outside the nanoscale range, up to one micrometer (1,000 nm) (FDA, 2014b). Compared to larger materials with the same elemental composition, ENMs may have enhanced or novel properties and may exhibit a wide variation in their structure as well as in their physical and chemical properties. These enhanced and novel properties of ENMs have led to their use in a broad range of fields, including agriculture (Adisa et al., 2018; Borgatta et al., 2018; Kah et al., 2019), consumer products^1^, environmental remediation (Petersen et al., 2012; Lowry et al., 2019; Zhang et al., 2019), food production and packaging (Uddin et al., 2016; Szefler, 2018), and nanomedicine (Besinis et al., 2015; Rösslein et al., 2017; Sun et al., 2017). Due to the widespread use of ENMs, it is necessary to ensure that potential environmental (Waissi-Leinonen et al., 2012; Edgington et al., 2014; Mortimer et al., 2016; Lead et al., 2018; Geitner et al., 2020) or human health (Nelson et al., 2013; Grafmueller et al., 2015; Fadeel et al., 2018; Salieri et al., 2020) risks of ENMs are understood and minimized.

In the United States, multiple federal agencies are tasked with the oversight and regulation of ENMs and applications of nanotechnology. The evaluation of potential ecological and human health effects of ENMs is challenging because of the nearly endless varieties of ENMs that can be synthesized in terms of shapes, sizes, surface coatings, and elemental compositions (Nel et al., 2013a,b; Zhao et al., 2019). In addition, toxicological effects for “the same” type of ENM can differ depending on synthesis methods, manufacturer/supplier performing the syntheses, and how each ENM is handled (Griffitt et al., 2008; Harper et al., 2008; Jeevanandam et al., 2018; Renero-Lecuna et al., 2019) and disposed of along its life cycle (Oischinger et al., 2019). The exponentially increasing number of potential ENMs and the possible differences in properties between the same types of ENMs makes the use of slow, expensive *in vivo* toxicity testing impractical (Nel et al., 2013a,b; Shatkin et al., 2016).

An alternative approach to *in vivo* toxicity testing, envisioned to be more efficient, predictive, and economical than using animals for evaluating the potential toxic effects of chemicals, was proposed by the U.S. National Research Council (NRC, 2007; Andersen and Krewski, 2009; Krewski et al., 2014). This approach uses *in silico*, *in chemico*, and *in vitro* methods, collectively known as new approach methodologies (NAMs), to inform pathway-based toxicities, hazard assessment, and, in some cases, to predict the level of toxicity. NAMs may be more effective than *in vivo* tests in providing mechanistic information on the potential biological effects of ENMs through adverse outcome pathways (AOPs). AOPs are frameworks to link biological events (often using data obtained with NAMs) to adverse effects, such as describing the relationship between protein alkylation and liver fibrosis (Gerloff et al., 2017) or the link between the altered transcriptional responses of acute phase response genes in lung tissue and nanoparticle-induced cardiovascular disease (Saber et al., 2014; Hadrup et al., 2020). While standardized test methods have been developed to measure potential toxicological effects, the behaviors of ENMs (e.g., the potential to agglomerate and settle out of suspension, or to react with test media and/or testing components) can challenge the performance of *in vitro* NAMs (Grieger et al., 2009; Kühnel and Nickel, 2014; Rösslein et al., 2015; Jeevanandam et al., 2018; OECD, 2018a). This has led to a sustained research effort to evaluate the applicability of test methods for use with ENMs and to design control experiments to test for potential biases and artifacts (Keene et al., 2014; Guadagnini et al., 2015; Hanna et al., 2018; Petersen et al., 2019b). However, it is not yet fully clear to what degree different U.S. regulatory agencies would accept results from standardized *in vitro* or *in chemico* NAMs and what methodological modifications are needed to yield robust, relevant results.

The Strategic Roadmap for Establishing New Approaches to Evaluate the Safety of Chemicals and Medical Products in the U.S.^2^, developed by the Interagency Coordinating Committee on the Validation of Alternative Methods (ICCVAM), guides activities to support the development of NAMs and increase confidence in their use among U.S. regulatory agencies. ICCVAM is composed of representatives from 17 U.S. federal agencies that use, generate, or disseminate toxicological and safety testing information^3^. The committee facilitates the development, validation, and regulatory acceptance of NAMs and other approaches that replace, reduce, or refine the use of animals for chemical safety testing^4^.

To perform specific tasks for the development or validation of NAMs, ICCVAM establishes *ad hoc* workgroups^5^. ICCVAM established its Nanomaterials Workgroup (NanoWG) to identify and evaluate ENM-specific testing requirements/recommendations among different U.S. government agencies, to determine whether ENM testing requirements/recommendations among the different agencies differ from testing requirements/recommendations specified for other types of substances, and to identify opportunities for NAMs to be used or developed to address agency needs.

This article summarizes the NanoWG’s evaluation of U.S. government agency requirements/recommendations for ENM testing. During this process, the NanoWG identified key considerations that need to be evaluated before NAM-based methods can be used to conduct safety testing on ENMs. Based on the information provided by the agencies on ENM-specific testing requirements/recommendations, we were able to collate references to published documentary standards that have been published relevant to ENM hazard testing. We also discuss key issues regarding control measurements and dosimetry during *in vitro* testing when evaluating ENMs. This article is not intended to be a comprehensive collection of all test methods used to evaluate ENM toxicity, nor is it a complete compendium of all U.S. agencies, offices, or divisions that utilize ENM testing. The article is intended to provide information to guide future discussion of approaches to advance the use of NAMs for evaluating the hazards of ENMs. Additional information on the regulatory framework for nanomaterials may be found in Ridge (2018), and a recent review by Shaffer et al. (2021) provides an overview of the agencies that perform chemical evaluations for different exposure scenarios.

## 2 Methods

The NanoWG surveyed ICCVAM member agencies to request information as to which ENMs are of agency interest, which toxicity tests were performed on ENMs to meet agency information requirements, and whether there are agency-specific guidance documents for ENM toxicity testing currently in place. Designated agency NanoWG representatives reviewed and compared their Agency’s current toxicity data requirements to generate responses and disseminated the survey information to appropriate staff members and other divisions for their input on needs and data challenges. Responses were received from the Centers for Disease Control and Prevention/National Institute for Occupational Safety and Health (CDC/NIOSH), U.S. Consumer Product Safety Commission (CPSC), U.S. Department of Defense, U.S. Department of Energy, U.S. Department of the Interior, U.S. Environmental Protection Agency (EPA), U.S. Food and Drug Administration (FDA), National Institute of Standards and Technology (NIST), and U.S. Department of Agriculture. These responses are summarized in Section 3. Some agencies responded that while they do not require or conduct toxicology testing, they are involved in the development and use of reference materials and standard methods related to ENM testing and evaluation. Tables 1, 2, and 3, respectively, include information on ENMs of agency interest, some test guidelines under which nanomaterials test data are submitted, and ENM-specific guidance documents developed by regulatory agencies.

As mentioned previously, differences in ENM synthesis and handling can alter their toxicological profiles. ENMs also tend to agglomerate/aggregate, settle out of suspension, or react with test media and/or testing components. Consequently, these properties indicate that ENMs have complex dosimetry, and therefore characterization of test media and/or testing components is a critical part of testing. The NanoWG also conducted an additional survey to discuss considerations for ENM characterization and dosimetry for *in vitro* assays. Responses were received from CPSC, EPA, FDA, and CDC/NIOSH, and are discussed in Section 4.

## 3 Agency needs for ENM testing

Agency responses regarding ENMs of interest, tests used to evaluate ENMs, and agency-specific guidance documents were compiled and reviewed and are discussed in more depth below. Agencies or divisions that have an interest in ENMs but do not require or conduct testing are the Pacific Northwest National Laboratory of the U.S. Department of Energy, the U.S. Department of the Interior, CPSC, and the U.S. Department of Agriculture National Institute of Food and Agriculture.

### 3.1 Responses relating to materials of interest

Given that the type of ENM and its end use may influence the required testing, the workgroup sought information about what ENMs are of interest to member agencies. The identified materials of interest are presented in Table 1, along with some of the use cases that brought these materials to agency attention. While a broad range of ENMs was represented in responses, almost all the most common ENMs are a focus for at least one agency. Some ENMs, such as carbon nanotubes, graphene family materials, metal oxides, nanoclays, and nanosilvers are a focus for multiple agencies. In addition to providing information about materials of interest, agencies also provided information on why the materials are of interest and indicated which materials are emerging concerns.

CPSC indicated that graphenes and nanoclays are emerging nanomaterials of interest, as well as complex mixtures of carbon nanotubes, metal ENMs, and other particles. They also stated that recently published studies have detected styrene, metals, and carbon nanotubes in the emissions from 3D printers, and carbon nanotubes, nanometals, metal oxides, polycyclic aromatic hydrocarbons, ozone, and carbon dioxide in emissions from laser printers (Kim et al., 2015; Pirela et al., 2019), and that these emissions will require further study.

EPA and CPSC collaborate to evaluate the potential release of free ion or micronized (e.g., formulations consisting of copper carbonate particles ranging in size from a few nanometers to several microns) copper particles from the paint or coating containing nanocopper and nanocopper pressure-treated lumber during their normal use, as well as to evaluate the effects of released metal oxides from treated wood. There is also a potential interest in other forms of nanocopper (e.g., aqueous alkaline copper azole), which has similar use applications and toxicological outcomes to micronized copper.

EPA’s Office of Pesticide Programs (OPP) indicated that nanosilica and nanometals bound to nanosilica and mixtures of nanometals were of emerging interest. EPA’s Office of Pollution Prevention and Toxics (OPPT) indicated that graphene and graphene oxides are emerging nanomaterials of interest.

FDA’s Center for Food Safety and Applied Nutrition (CFSAN) commented that while nanoclays are used in food packaging, they are not expected to migrate into food products. There is potential dietary exposure to ionic copper or silver derived from food contact packaging use of nanoparticulated silver or copper. Because titanium dioxide and silicon dioxide, when used as direct food additives, may contain some particles in the nanoscale range, consumers may also be exposed to nanoparticulate forms of titanium dioxide and silicon dioxide.

CDC/NIOSH’s Nanotechnology Research Center (NTRC) is the leading federal agency conducting research and providing guidance on the occupational safety and health implications and applications of advanced materials and nanotechnology. NTRC has a robust field study and laboratory research program that investigates ENM toxicity and conducts exposure assessments and epidemiological studies in the workplace. In addition, the NTRC focuses on critical areas of ENM research including material properties such as dustiness and explosivity behavior, and emissions characteristics of nanomaterials and NM-enabled products that are important in assessing potential toxicity and risk associated with real-world occupational exposures (Bishop et al., 2017). The data suggests that low solubility nano-scaled particles are generally more toxic than larger particles on a mass-to-mass basis (Oberdörster et al., 2005; Rothen-Rutishauser et al., 2007; Sager and Castranova, 2009; Zhao et al., 2009; Bakand et al., 2012). There are also strong indications that particle surface area, surface chemistry, and solubility play a role in the observed toxicity of ENMs in cell culture and animal models (Sager and Castranova, 2009; Roberts et al., 2013). *In vitro* models employing both acute and sub-chronic exposure conditions have been developed and used to predict *in vivo* toxicological responses (Cho et al., 2013; Manke et al., 2014; Wang et al., 2014). Based on comparable exposure doses, time courses, target cell types, and relevant biological endpoints, consistent results have been obtained from comparable experiments with *in vitro* vs. *in vivo* models using similar ENMs (e.g., based on physicochemical properties) such as carbon nanotubes (Mercer et al., 2011; Mishra et al., 2012; Sargent et al., 2014; Siegrist et al., 2014; Snyder-Talkington et al., 2015, 2019), metal oxide nanoparticles (Ma et al., 2015; Davidson et al., 2016), boron nitride nanotubes (Kodali et al., 2017; Xin et al., 2020), and end-life cycle (incinerated) nanoclay enabled thermoplastics (Stueckle et al., 2018; Wagner et al., 2018). These results, mainly observed from CDC/NIOSH research projects on the ENMs of agency interest listed in Table 1, support the implementation of *in vitro* models as a rapid and economical tool to screen and predict the potential *in vivo* toxicological responses to ENMs for reducing, refining, and replacing animal usage.

The U.S. Department of Agriculture Forest Service Forest Products Laboratory is primarily or partly responsible for the development of many of today’s wood-based technologies such as wood science, building structures, building resilience, building materials, pulp and paper, biofuels, performance polymers from wood, and high-value chemicals from wood. In the area of nanotechnology, the laboratory focuses on research into the application of cellulose ENMs, the nanoscale aspects of wood, especially renewable, forest-based nanomaterials, and partners with other organizations on understanding the environmental, health and safety aspects of forest-based nanomaterials.

### 3.2 Responses relating to methods and guidance documents relevant to ENM toxicity testing

One difficulty with the evaluation of ENMs is determining when ENM-specific testing is required. For example, an agency’s definition of what may be considered an “ENM” varies between U.S. agencies and may be dependent on end use. This implies the need for a case-by-case ENM-specific safety assessment, based on the material’s characteristics, the proposed use of the material, and the route of exposure/administration, among other factors (FDA, 2014b; EPA, 2017). As described in Table 1, there are multiple types of ENMs of interest to U.S. agencies, spanning an array of applications and uses. While testing of ENMs often needs to be evaluated on a case-by-case basis, there are test guidelines, provided in Table 2, that are frequently used for the evaluation of ENMs for use as food additives, new dietary ingredients, pesticides, or as part of pesticide formulations. Table 2 is not intended to be a complete compendium of all test methods used to evaluate ENM hazard, nor should it be implied that these guidelines are only used to test the substances/products indicated in the table. In addition to the guidelines listed in Table 2, some agencies (such as EPA) allow studies to be conducted in accordance with Organisation for Economic Co-operation and Development (OECD) guidance^6^. Moreover, EPA requires or recommends that protocols be submitted prior to study submission if modifications of these methods are proposed for toxicity testing of ENMs. It was often not possible to provide prescriptive suggestions about what specific methods are acceptable for testing ENMs, because the science on this topic is rapidly evolving and decisions are often made on a case-by-case basis. Given this rapid evolution, consensus has not yet been reached within agencies on some topics.

EPA OPP regulates the manufacturing and use of pesticides (including insecticides, herbicides, rodenticides, disinfectants, sanitizers, etc.) in the United States and establishes maximum levels for pesticide residues in food. OPP operates under the Federal Insecticide, Fungicide, and Rodenticide Act (FIFRA), which governs pesticide registration, distribution, sale and use. Enacted in 1947, FIFRA sets risk/benefit standards for pesticide registration, requiring that pesticides perform their intended function, when used according to labeling directions, without posing unreasonable risks of adverse effects on human health or the environment (7 U.S.C. § 136 et seq., 1947). In 1972, FIFRA was amended, expanding EPA’s authority to strengthen the registration process, enforcement provisions, and broaden the legal emphasis on further protecting health and the environment (7 U.S.C. § 136 et seq., 1972). FIFRA was further amended by the Food Quality Protection Act (FQPA) (7 U.S.C. §136, 1996) and the Federal Food Drug and Cosmetic Act (21 U.S.C. §301 et seq, 2002), under which EPA establishes tolerances or maximum legal limits for pesticides that apply to food. Under FQPA, a collection of pesticide data is necessary to set allowable levels and to conclude that a pesticide is safe. The rule further ensures that no harm will result to infants and children from aggregate exposure to the pesticide chemical residue. As a result, pesticide products, including ENM-containing antimicrobial products, inquiring registration require various data generation to address potential adverse effects to humans and environmental fate.

In evaluating a pesticide registration application, OPP assesses a wide variety of potential toxicological effects associated with the use of the product or active ingredient. In general, for ENMs, OPP requires data generated with the toxicological test guidelines presented in Table 2, but ENMs’ physical-chemical product characteristics are evaluated by product chemistry test guidelines and often compared with ENMs reported in toxicology studies.

EPA OPPT administers the Toxic Substances Control Act (TSCA; (15 U.S.C. §2601 et seq., 1976)), which regulates chemical substances and mixtures that are manufactured, imported, processed, distributed, used or disposed of in the United States and that are not regulated under other laws (such as those that apply to pesticides or food and drugs). TSCA was originally enacted in 1976 and serves as the nation’s primary chemicals management law. In 2016, TSCA was amended by the Frank R. Lautenberg Chemical Safety for the 21^st^ Century Act, which included language to encourage alternatives to animal use for testing done under TSCA (15 U.S.C. § 2601 et seq., 2016).

Under TSCA, most nanomaterials are regarded as “chemical substances”. New chemical substances manufactured at the nanoscale must be submitted to EPA review before they can enter the marketplace^7^. Although upfront toxicity testing is not required under TSCA for any chemical substance, including ENMs, manufacturers must submit any existing data in their possession or control at the time of the new chemical application in a premanufacture notice. Premanufacture notice submissions for new nanomaterials under TSCA are reviewed and regulated individually. If EPA determines that the available information is insufficient to make a reasoned evaluation as to whether an ENM might produce an unreasonable risk to human health or the environment under the expected conditions of use, the agency may issue a consent order under Section 5(e) of TSCA to the submitter for additional testing. The recommended testing is specific to the area of human health concern. For example, if the concern is about inhalation exposure to various nanoparticles, the recommended testing may include an inhalation toxicity study (OPPTS Test Guideline 870.3465 (EPA, 1998f) or OECD Test Guideline 413 (OECD, 2018b).

The 2016 Lautenberg Chemical Safety Act (15 U.S.C. § 2601 et seq., 2016) requires EPA to develop a plan to “*promote the development and implementation of alternative test methods and strategies to reduce, refine, or replace vertebrate animal testing and provide information of equivalent or better scientific quality and relevance for assessing risks of injury to health or the environment of chemical substances or mixtures*.” As part of this effort, EPA published a strategic plan in 2018 (EPA, 2018) to promote the development and implementation of alternative test methods or NAMs and a list of acceptable NAMs within the TSCA program (EPA, 2021). Even though NAMs presented in this list are not specific to ENMs, EPA expects to consider NAMs for several TSCA ENM decision contexts including hazard identification and characterization.

Table 3 lists selected guidance documents that U.S. federal agencies have issued to advise stakeholders on ENM testing. In 2017, EPA issued guidance (Tab. 3) to assist companies to report under the TSCA nanotechnology reporting and recordkeeping requirements rule (EPA, 2017). This rule mandates that manufacturers report information including specific chemical identity, production volume, methods of manufacture and processing, exposure and release information, and existing data on environmental and health effects.

FDA recently released a progress report (FDA, 2020) that shows a steady increase in drug product submissions containing nanomaterials to FDA. These submissions include nanomaterials of differing compositions, sizes, and surfaces, as well as nanomaterials containing therapeutic agents (Farjadian et al., 2019). FDA has issued several guidance documents on topics related to the application of nanotechnology in FDA-regulated products (Tab. 3) as part of ongoing implementation of recommendations from FDA’s 2007 Nanotechnology Task Force Report (FDA, 2007). These documents serve to convey FDA’s current opinion on a topic rather than to bind the FDA or the public.

In 2014, FDA issued the FDA Final Guidance for Industry — Considering Whether an FDA-Regulated Product Involves the Application of Nanotechnology (FDA, 2014b). This guidance describes an overarching framework for FDA’s approach to the regulation of nanotechnology products. FDA has not established a regulatory definition of nanotechnology, nanomaterial, nanoscale, or related terms. In this overarching guidance, FDA identified two “points to consider” that should be used to evaluate whether FDA-regulated products involve the application of nanotechnology:

1. Whether a material or end product is engineered to have at least one external dimension, or an internal or surface structure, in the nanoscale range (approximately 1 nm to 100 nm);
2. Whether a material or end product is engineered to exhibit properties or phenomena, including physical or chemical properties or biological effects, that are attributable to its diniension(s), even if these dimensions fall outside the nanoscale range, up to one micrometer (1,000 nm).

The FDA Center for Devices and Radiological Health follows this guidance when evaluating new medical devices. A key statement from this document is: “*Based on our current scientific and technical understanding of ENMs and their characteristics, FDA believes that evaluations of safety, effectiveness, public health impact, or regulatory status of nanotechnology products should consider any unique properties and behaviors that the application of nanotechnology may impart*.”

In addition to the FDA Final Guidance for Industry – Considering Whether an FDA-Regulated Product Involves the Application of Nanotechnology, the Center for Drug Evaluation and Research also refers to another draft guidance^8^, Drug Products, Including Biological Products, that Contain Nanomaterials – Guidance for Industry (FDA, 2017). This draft guidance “*does not address, or presuppose, what ultimate regulatory outcome, if any, will result for a particular drug product that contains nanomaterials*.” Safety, effectiveness, public health impact, and regulatory status of drug products that contain ENMs are currently addressed on a case-by-case basis using FDA’s existing review processes. Current Center for Drug Evaluation and Research guidance documents and requirements for the evaluation and maintenance of quality, safety, and efficacy apply to drug products containing ENMs that fall within their scopes. “*As such, this guidance should be viewed as supplementary to other guidances for drug products*” (FDA, 2017).

FDA has also issued guidance documents pertaining to ENMs in food (FDA, 2014a). CFSAN has premarket authorization authority over food additives and new dietary ingredients under the United States Federal Food, Drug, and Cosmetic Act (21 U.S.C. §301 et seq, 2002). As both product areas concern potential oral exposure to an ENM, the toxicity testing paradigms generally used to evaluate the safety of food additives or new dietary ingredients primarily comprise repeated oral dosing studies in rodents. Existing test guidelines describing repeated oral dosing and inhalational exposure studies in rodents (EPA, 1998e,f; OECD, 1998b; EPA, 2000; OECD, 2008, 2009b, 2018b) appear to be appropriate for use with ENMs (OECD, 2009a, 2012).

To evaluate carcinogenicity of these products, genotoxicity studies, such as the Ames assay or the mouse lymphoma assay, are used to ascertain the mechanism of action of any observed neoplastic effects in rodent bioassays (Kobets et al., 2018). However, for the Ames assay, some ENMs have been shown to be unable to enter the bacterial cells, which would make such test articles incompatible with the test system (Woodruff et al., 2012). It is notable that none of the standard OECD test guidelines on *in vitro* genotoxicity assays has been validated for use with ENMs, though the guideline describing the *in vitro* mammalian cell micronucleus test directly acknowledges the requirement for methodological adaptation for ENMs (OECD, 2016). In addition, toxicokinetic studies may be used to inform the safety assessment regarding the potential for systemic exposure to the food additive or new dietary ingredient, for route-to-route extrapolation from the results of non-oral toxicity studies, and for refining the inter- and intraspecies uncertainty factors used in quantitative risk assessment for non-neoplastic endpoints.

CDC/NIOSH leads the federal government health and safety initiative for nanotechnology^9^. Research and activities are co-ordinated through CDC/NIOSH’s NTRC. The contributions of NTRC to the nanotechnology and nanotoxicology fields include the guidance documents of safety programs, guidelines, and design solutions for ENM workplaces (Tab. 3).

The CPSC’s regulations do not require testing; the Federal Hazardous Substances Act (15 U.S.C. §1261 et seq., 2008) and its implementing regulations only require that a product be labeled to reflect the hazards associated with that product. Manufacturers, retailers, and distributors of nano-enabled products, as with any consumer product under the CPSC’s jurisdiction, must report to the CPSC immediately if they obtain information that reasonably supports the conclusion that their product fails to comply with an applicable consumer product safety rule, contains a defect that could create a substantial product hazard, or creates an unreasonable risk of serious injury or death (CPSC, 2019).

The U.S. Department of Defense generally uses data collected using EPA’s guidelines for ENM testing. Some specific tests such as the zebrafish (*Danio rerio*) embryo test (OECD, 1998a; Haque and Ward, 2018) or *Daphnia magna* toxicity testing (Xu et al., 2019) are primarily directed at understanding the ecotoxicity of novel ENMs.

In addition to the test guidelines and guidance documents identified in Tables 2 and 3, the NanoWG compiled a list of documentary standards and guidelines designed or evaluated for ENM characterization and/or toxicity testing issued by the American Society for Testing and Materials International (ASTM), the International Organization for Standardization (ISO), and the OECD Working Party on Manufactured Nanomaterials. The compiled list, which contains recommended vocabularies for ENMs, methods for the characterization of ENMs, and some methods for working with and evaluating ENMs, is presented in Table S1^10^. This compilation of methods has been prepared to support scientists with identifying potentially relevant standards. While some of these methods describe toxicity tests designed for use with ENMs (e.g., ASTM E2526 (2013)), many also describe the protocol considerations and measurements that are needed to support toxicity testing such as ENM characterization in the test media and quantification of the ENM concentration. The key issue of dosimetry during *in vitro* tests with ENMs will be discussed in depth in Section 4.2.

## 4 Practical considerations for *in vitro* toxicity testing of ENMs

Compared to substances that readily dissolve in test medium or other solvents, ENMs pose multiple challenges owing to their unique physicochemical characteristics. It is increasingly realized that commonly used *in vitro* inhalation toxicity study models where the effects of ENMs on cultured cells are tested under submerged conditions, may not represent real exposure conditions, i.e., inhaled “dry” ENM deposition in the lung. One of the foremost challenges in ENM testing relates to changes in dosimetry occurring during experiments (Teeguarden et al., 2007; DeLoid et al., 2017). Changes in dosimetry can occur as a result of each ENM’s effective density in culture medium (DeLoid et al., 2014; Pal et al., 2015), dissolution of particles (e.g., nanosilver particles dissolving and forming silver ions (Liu et al., 2010)), agglomeration of particles (e.g., particles interacting with other particles to form larger agglomerates (Li et al., 2010)), heteroagglomeration of the particles (e.g., particles interacting with, for example, algae or bacterial cells during the assay to form agglomerates (Hartmann et al., 2012; Hanna et al., 2018)), and transformations such as redox changes (e.g., changes in the speciation of particles such as the conversion of AgNPs to silver chloride particles (Ha et al., 2018; Poli et al., 2020)). Dissolution, agglomeration, and/or redox changes can cause the exposure concentration to vary substantially when testing pelagic organisms (i.e., organisms in the water column such as Daphnia magna) or suspended cells. In addition, the results of *in vitro* assays for some ENMs may vary strongly based on the composition of the test medium, which can impact the dissolution of ENMs, their transformations (e.g., redox changes), or the formation of a protein corona (Drasler et al., 2017; Kaiser et al., 2017). Another key challenge that we discuss in Section 4.3 is the potential for experimental artifacts during toxicity testing of ENMs. This necessitates adequate control experiments to identify and minimize potential artifacts and may reveal additional control experiments required for elucidating mechanisms of toxicity.

One approach that may have more physiological relevance and overcome some of the issues with transformations that can occur during exposure with submerged models is to expose cell culture models having an air-liquid interface to aerosolized ENMs. This exposure approach utilizes cells grown on porous culture inserts, such as 3D models with pseudostratified epithelium and intact mucosa and cilia, which enables direct deposition of nanoparticle powders through aerosol exposure. This approach has been used in numerous recent ENM studies (Polk et al., 2016; Drasler et al., 2017; Barosova et al., 2020; Leibrock et al., 2020).

### 4.1 Dosimetry survey responses

The complexity of ENM dosimetry (i.e., particle agglomeration/aggregations, redox changes, interaction of particles with proteins in media, particle dissolution rate, etc.) led the NanoWG to develop a list of detailed considerations for those using *in vitro* tests (Tab. 4). The measurements in Table 4 are suggested based on best practices from the scientific literature. However, it is important to note that standardized methods are not yet available for some potential dose metrics such as particle number concentration or surface area concentration. Additional concerns are described below.

Accurate dosimetry measurements, in general, are challenging and may not be technically feasible for all types of ENMs (Johnston et al., 2020). For example, it is substantially more difficult to characterize the agglomeration status of rod- or plate-shaped ENMs than that of spherical nanoparticles. This is because dynamic light scattering, a commonly used agglomeration characterization method, typically determines the hydrodynamic diameter of an ENM based on the size of a sphere that diffuses at the same rate as the particle being measured (Petersen and Henry, 2012; Carvalho et al., 2018). In addition, commonly used *in vitro* dosimetry models for submerged cells are limited to relatively low-aspect-ratio ENMs (i.e., those with a length similar to their width) (DeLoid et al., 2017).

Another factor that must be accounted for is the effective density of the ENM agglomerate unit, which includes both the particles and the media (DeLoid et al., 2014). The effective density for an ENM can vary greatly from one culture medium to another, thus changing the delivered dose to the cells for the same ENM. The capacity to characterize different concentration dose metrics also varies based on the type of ENM and its agglomeration state (Minelli et al., 2019). For example, a comparison of the number concentration measurements of gold ENMs had substantially worse agreement among techniques for samples which showed substantial agglomeration than for those that remained individually dispersed (Petersen et al., 2019a). The detection limit of analytical methods to quantify ENM mass concentration in test media for *in vitro* NAMs also varies for different ENMs. For example, it is difficult to measure the concentration of carbonaceous ENMs in test media with high concentrations of serum (Petersen et al., 2016; Goodwin et al., 2018), while the presence of serum in medium is less problematic for quantification of metal and metal oxide ENMs (Laborda et al., 2016).

The procedure to prepare an ENM suspension at the necessary concentration prior to an assay can vary greatly among laboratories, which may change the experimental outcome. Thus, there is a need to standardize the preparation for each ENM to reduce variability between testing laboratories. For example, most ENMs are sonicated prior to testing, but the level and duration of the sonication can vary, which affects the amount of energy delivered to the material. This variation can affect the agglomeration size, which ultimately affects the dose of material delivered to the cells. A way to minimize variation is to calorimetrically calibrate all sonicators to ensure the exact same energy is delivered to the material each time for consistent dispersion results (Taurozzi et al., 2011). Also, the total delivered sonication energy and the number of sonications needed to disperse ENMs should be reported for each study.

Table 4 was circulated within the workgroup to assess the relevance of these considerations on the characterization of ENMs to agencies’ information needs. As expected from agencies with very different testing needs, responses to Table 4 varied.

Responses from EPA OPP were that several characterizations (i.e., ENM mean size prior to addition to test media, ENM size distribution prior to addition to test media, ENM mean size in test media prior to exposure period, ENM size distribution in test media prior to exposure period, and ENM dissolution in test media before and after exposure period) are not required as part of toxicity testing, but are requested as part of physicochemical properties of products and environmental fate determinations. Thus, these measurements are not necessarily made in the presence of cell culture or environmental media. ENM mass concentration in test media before exposure period are not required, but OPP typically requests clarification of such information as part of the dissolution kinetic studies when test media are buffer solutions or water. For toxicology studies, if not provided, OPP encourages registrants “*to provide nanomaterial mass concentrations in media*” under certain circumstances. It is important to note that, if ENM-specific modifications to test methods are needed, a revised protocol submission is recommended for review prior to initiating the study. Such modifications may be needed to generate robust results.

EPA OPPT stated that manufacturers are not required to submit any specific dosimetry characterization data for ENMs. However, manufacturers are encouraged to submit ENM mean size and size distribution before exposure period along with other standard physicochemical characterization data, which may assist with EPA’s understanding of the toxicity of an ENM.

For review of engineered nanomaterial food contact substances where consumer exposure to the nanomaterial is expected, FDA CFSAN requires the following ENM-specihc information: particle number or surface-area concentration in test media before exposure period, ENM mean size or size distribution prior to addition to test media, and ENM mean size in test media prior to exposure period (Rice et al., 2009). ENM dissolution in test media after exposure period would be considered a key metric both in assessing test system exposure to the ENM and also in assessing the feasibility of using “read-across” to its non-nano analogs (e.g., a particle with the same composition and shape with all dimensions > 100 nm) in the safety assessment of the ENM. CFSAN indicated that some information such as measurements or modeling of ENM mass concentration associated with cells after the exposure period would be considered key metrics for documenting exposure of the test system to the test article.

Regarding delivered dose, there was discussion about the benefits and limitations of two different particokinetic models: the *in vitro* sedimentation, diffusion, and dosimetry (ISDD) model (Hinderliter et al., 2010; DeLoid et al., 2017) and the *in vitro* sedimentation diffusion, dissolution, and dosimetry (ISD3) model (Thomas et al., 2018). NanoWG discussion specifically concerned the models’ usefulness in relating a nominal concentration to an estimate of the actual amount of ENMs reaching the cells. Ultimately, the workgroup reached no consensus as to how to use different dose metrics or particokinetic models to understand the results from *in vitro* studies, although several workgroup members agreed with the CPSC response that, in general, robust studies include hydrodynamic or aerodynamic size distribution data for aqueous dispersions or airborne ENMs before the start of the exposures.

The measurements presented in Table 4 are not necessarily required data for the submission of ENMs to regulatory agencies, and there is still debate within and across agencies as to which data should be required or considered as part of toxicity study requirements for ENMs. Nonetheless, the measurements are still useful for consideration during the development and testing of ENMs.

### 4.2 Dosimetry considerations

Table 5 lists five main categories of *in vitro* test exposure systems. The choice of whether to require additional dosimetry measurements for *in vitro* methods may vary based on the exposure system used.

While promising research has been conducted on the fourth (airborne exposure to a biological test system located on an air-liquid interface (Lacroix et al., 2018; Barosova et al., 2020)) and fifth (lung-on-a-chip model of inhalation toxicity (Zhang et al., 2018)) exposure systems/categories, there are no standardized methods using these exposure approaches. Thus, this discussion will focus on the first three types of exposure systems.

As described in Section 4.1, dosimetry and dosimetry requirements/recommendations for ENMs can be complex, differing to some extent among agencies, and detailed guidance is not always available. In the absence of such guidance, it can be helpful to consider the dosimetry requirements for testing dissolved substances, which are described in detail for the OECD testing program. For human health testing for either *in vivo* or *in vitro* measurements, only the verification of the initial dose is required. However, it is widely known that the exposure concentration of dissolved chemicals can vary due to factors such as volatilization, adsorption to the well sidewalls, and metabolism (Tanneberger et al., 2013). The trade-offs between test method accuracy and the additional costs and workload associated with testing the concentrations in the wells is a topic of ongoing discussion (Natsch et al., 2018). In addition, numerous efforts have been made to move from a nominal to a cellular concentration in *in vitro* assays using submerged culture exposure conditions and in associated *in vitro* to *in vivo* extrapolation modeling (Amritage et al., 2014; Casey et al., 2018).

Nominal concentrations are typically used for *in vitro* measurements for human health endpoints, which raises questions about the dosimetry requirements for *in vitro* tests of ENMs and whether it is justified to require more detailed information for the dosimetry of ENMs than for other test substances. OECD GD 317 (2020) addresses dosimetry concerns for aquatic toxicity testing of ENMs and may provide guidance on how to handle exposure measurements for *in vitro* testing for human health testing requirements if additional dosimetry measurements are deemed necessary. Multiple dose metrics are considered: mass concentration, nanoparticle number concentration, and surface area concentration, all of which have been successfully used in the published literature. However, as stated above, there is a lack of standardized methods for measuring the nanoparticle number and surface area concentrations. Recent studies have shown substantial differences in the nanoparticle number concentration among techniques (Amini et al., 2016; Mourdikoudis et al., 2018; Petersen et al., 2019a). Thus, this guidance document suggests that mass concentration measurements should be required, although additional ENM characterization and dosimetry measurements in the test media can also be provided.

In NAMs with liquid exposure to suspended molecules or cells (Category 1 of Tab. 5), rapid agglomeration and settling of the ENM in these systems would reduce the suspended exposure concentration to the ENM. Therefore, it may be appropriate to measure the change in the suspended ENM mass concentration across the duration of the assay to evaluate if the concentration is constant, unless the ENM concentration at the bottom of the test container would be expected to have the same effect as the fraction that remains suspended. For Category 2 assays, those in which cells growing in monolayers are submerged in media, it is possible to quantify changes in the suspended concentration during the exposure period and to estimate that the exposure concentration is equivalent to the change in the suspended concentration. For the third exposure approach (Category 3: a liquid, cream, or solid directly applied to a biological test system such as a 3D construct), determining the ENM mass applied to the surface is likely sufficient. The exposure concentration on the biological test article can be determined from the ENM concentration in the formulation or solid and the mass or volume applied to the biological construct. For submerged cell model exposure (Category 2), there have also been extensive efforts to model the expected cellular exposure concentration based on the effective density and size of the ENM, as described above for the ISDD model (Hinderliter et al., 2010; Thomas et al., 2018). However, this approach has not yet been standardized, the reproducibility of effective density measurements has not undergone interlaboratory testing, and the modeled cellular concentration may depend upon the method used to quantify the ENM size (Petersen et al., 2019a). Further dosimetry modelling to model deposition relies upon accurate input parameters, such as dispersant density and viscosity, that are not universally available. This can lead to uncertainty in attaining expected cellular exposure concentrations; therefore, in the absence of parameters published in the literature, the required parameters should be experimentally derived. Lastly, gaps in dosimetry include the impact of physiochemical parameters on ENM behavior in medium during dosing, modeling deposition within the cellular environment for high-aspect ratio fibers (Price et al., 2019) and two-dimensional ENMs, and efficient dosing with buoyant ENMs, such as virgin and nano-enabled composite thermoplastics. Until robust models are developed and validated, secondary analytical techniques presented in Table 4 should be considered to reduce uncertainty in assessing cellular exposure.

### 4.3 Interference/bias controls

One of the foremost challenges in using *in vitro* test methods with ENMs is the potential for analytical biases or artifacts (i.e., problems that occur during the test leading to an incorrect result or misinterpretation). *in vitro* ENM studies often either overlook or provide incomplete interference characterization (Ong et al., 2014), because control experiments to detect and characterize ENM-derived artifacts are often not performed. A list of potential control experiments is provided in Table 6 along with assays that could be impacted by each artifact. No specific recommendations or guidelines for the detection and characterization of method specific ENM interference currently exist. Since each test method is performed under a unique set of circumstances, which may include method-specific reagents, incubation temperature and times, or biological sample matrices, it is necessary to critically review each parameter prior to determining what control experiments may be needed when testing a particular ENM.

If artifactual results are expected or observed, it may be necessary to consider whether mitigation strategies, bias characterization, or complete methodological replacement are warranted. In the case of cytotoxicity, membrane integrity, and proliferation screening assays, no single method is universally robust against interference for all ENMs (Monteiro-Riviere et al., 2009; Kroll et al., 2012). Therefore, each ENM-method pairing should be screened for known sources of interference highlighted in Tables 6 and 7 to determine analytic fitness for purpose and to characterize approximate direction and magnitude of analytic bias, if possible (Han et al., 2011; Holder et al., 2012). In addition to the sources of interference highlighted in the tables, when using methods with indirect measurement endpoints, e.g., colorimetric, fluorometric, luminometric, etc., ENM absorbance, quenching, and autofluorescence should be examined to assess appropriateness of that method. Where applicable, signal inhibition/enhancement and spike-in control experiments may be warranted. Further, measures of cytotoxicity, membrane integrity, and proliferation can be performed using two or more concurrent methods to assess concordance and facilitate result interpretation.

In certain instances, method replacement may not be plausible, and adaptation of an extant method may be required. Here, we use the *in vitro* cytokinesis-block micronucleus assay using cytochalasin B, which is a standard assay for measuring genotoxicity of a chemical (Fenech, 1997), as an example. In the method, cytochalasin B is added to cultured cells to inhibit cytokinesis, but it also inhibits actin assembly, which can decrease cellular uptake of ENMs (MacLean-Fletcher and Pollard, 1980; Kettiger et al., 2013). Therefore, while not formally adopted, the OECD has proposed methodological adaptation through delayed cytochalasin B treatment after ENM treatment to mitigate potential ENM uptake inhibition for the *in vitro* cytokinesis-block micronucleus assay (Gonzalez et al., 2011).

Under certain circumstances, artifactual influences on the biological system may be unavoidable. For example, the formation of a proteinaceous ENM corona can lead to immunomodulatory or toxicodynamic effects on *in vitro* models (Mo et al., 2018). Effects caused by a protein corona during *in vitro* experiments may not necessarily be translatable to *in vivo* models or the human milieu, but they cannot be immediately discounted given that the incorporation of nano-enabled medicines may potentially lead to bioavailable serum-bound ENMs (Rampado et al., 2020).

In addition to potential analytical artifacts and biases, it is possible to perform additional control experiments to better understand and contextualize the mechanism of toxicity to match inherent properties of a particular ENM and its respective exposure conditions. For example, the addition of a particle dispersant may impart a biological or toxicodynamic effect on the *in vitro* system that may not translate to the *in vivo* milieu. Though such controls are typically routine, the potential biological effects due to corona formation in the presence of proteinaceous dispersants, such as serum, should be considered. The toxicodynamic effects of dissolvable ions from ENM and leachable constituents from complex mixtures may warrant investigation with a myriad of methods, including treatments with soluble ion controls and filtrate controls. A list of experiments to understand the mechanism(s) of toxicity is presented in Table 7. For some contexts of use, gaining insight into the toxicity mechanism as well as contributory sources of biological effect may be critical for risk assessment, while for other contexts of use, this infonnation may not be essential but assists in interpreting the assay results. When conducting assays to fulfil regulatory requirements/recommendations, the relevant regulatory agency should be consulted to determine what control experiments are required prior to the submission of *in vitro* toxicity or efficacy test data.

## Conclusions and future directions

The NanoWG surveyed ICCVAM member agencies to request information as to which types of ENMs are of agency interest, which toxicology tests are performed on ENMs, whether there is agency-specific guidance for ENM toxicity testing, and what dosimetry and interference/bias controls are requested for the use of *in vitro* test methods with ENMs. Based on the responses received, the workgroup determined that there are significant challenges in identifying and clarifying the toxicity testing needs of ENMs across agencies and programs, because the requirements or key considerations at each agency differ based on the products they regulate. Therefore, the NanoWG evaluated two key issues, namely dosimetry and interference/bias controls, which are relevant across a broad range of NAMs when testing ENMs to assist *in vitro* method developers in understanding the perspectives of different agencies on these topics and to help provide general guidance.

Demonstrating the technical reproducibility and biological relevance of NAMs is the key to supporting their broader use for dissolved and particulate substances such as ENMs. One important topic for future work related to technical reproducibility to support the broader use of *in vitro* test methods is to provide clear guidance on determining whether a particular method is applicable for use with ENMs. This may require performing the assay with a specific set of ENMs with diverse properties such as different surface charges, elemental compositions, and surface coatings, and clarifying specific control measurements that should be performed simultaneously. If control measurements of a NAM show artifactual results with some types of ENMs, the applicability domain of the NAM may be limited to those ENMs that do not produce such results, or modifications to the NAM to minimize the effect of the artifacts may be needed.

An important topic for future work related to biological relevance is how to correlate *in vitro* and *in vivo* test results, and how to evaluate to what extent *in vitro* responses can be used to predict corresponding *in vivo* exposures and effects. This is especially important if the *in vitro* test results will be used for more than just screening and prioritization. As described in the ICCVAM roadmap (2018), it is recommended, when possible, to discuss proposed applications of NAMs with regulatory agencies during the NAM development process to carefully clarify the context of use. To validate the *in vitro* to *in vivo* correlation, it would be helpful to collect high-quality data available for different standardized *in vivo* test methods with different ENMs. These results could then be compared to those obtained using individual NAMs (e.g., lung fibrosis (Barosova et al., 2020)) or combinations of NAMs (e.g., those for skin sensitization (OECD, 2021b)) testing specific key events along an adverse outcome pathway (Halappanavar et al., 2019, 2020). Suggested priority areas for comparing *in vivo* results and NAMs are for endpoints that have demonstrated defined approaches (e.g., skin sensitization) for dissolved chemicals and for endpoints that have robust *in vivo* datasets with ENMs.

Stakeholders place confidence in data from toxicology test methods, i.e., that they are producing the correct result and identifying a potential hazard (or not). Hazard evaluation has historically been accomplished through *in vivo* approaches. As highlighted above, to establish confidence in NAMs, we compare them to the *in vivo* test method result, and discordance is viewed as a limitation of the NAM. However, in addition to assessing NAM reproducibility, several studies are now investigating the reproducibility of *in vivo* methods so that limitations can be taken into consideration in the context of any discordance noted when comparing to NAMs (Luechtefeld et al., 2016; Pham et al., 2020; Rooney et al., 2021). Other recent work has focused on evaluating traditional *in vivo* toxicity tests, as well as NAMs, based on their relevance to human biology (Clippinger et al., 2021). With that in mind and given the challenges to implementation of NAMs as complete replacements of animal use for testing single chemicals, it stands to reason that their implementation for testing ENMs has yet to be realized. Therefore, while substantial progress has been made in the testing of ENMs during the past two decades, additional work on these topics is needed to support the increased usage of *in vitro* test methods with ENMs for regulatory testing. Progress towards this goal will be predicated on federal agencies and stakeholders working together using flexible, robust, and integrated approaches to implement NAMs that both protect human and environment health and reduce or eliminate the need for testing in animals.

## Supplementary Material

BD5FA2B27A44129C49353F68BEC5AEB6

## Figures and Tables

**Tab. 1:** Examples of ENMs of agency interest

Agency	Material	Application/uses
CPSC	Carbon nanotubes	Batteries, fabrics, films, composites/coatings, electronics, filtration, inks and filaments, sensors
	Complex mixtures of carbon nanotubes, metal ENMs, and other non-nano materials	3D-printing and laser printer emissions
	Fullerenes	Batteries, cements, ceramics, coatings, electronics, flame retardants, glass, inks, paints, plastics, rubber
	Graphene	Filters/sorbents, surfactants/lubricants, batteries, lighting, electronics, coatings, fabrics, rubber products, inks, sensors
	Metal oxides (e.g., ZnO, CeO_2_, Fe_3_O_4_, TiO_2_)	Coatings for paint and wood treatments, fuel cells, abrasives, sensors, magnetic coatings, conductive films, composites
	Nanoclays	Adhesives, ceramics, coatings, cleaners, flame retardants, inks/pigments
	Nanosilicates	Cement, paints, adhesives, rubber, coatings, sensors
	Nanosilver, micronized copper	Textiles, cleaners, paints and coatings, sealants, filters, conductive inks
EPA Office of Pesticide Programs	Micronized copper	Paints and coatings, pressure-treated lumber
	Nanosilver	Textiles, plastic films, coatings, adhesives, pool treatments
	Nanosilica (Nanometals bound to silica or nano-sized silica)	Textile treatments and possible nanocarriers
	Metal oxides	Material preservatives, and possible photocatalytic device uses^a^
	Nanocopper^a^	Possible wood treatment uses, possible paint uses
	Mixtures of nanometals^a^	Possible glass implementation
EPA Office of Pollution Prevention and Toxics	Carbon nanotubes	Conductive plastics, batteries, flow and fuel cells, composite materials, flat-panel displays, micro- and nanoelectronics, ultra-capacitors, atomic force microscope tips, biosensors
	Graphene and graphene oxides	Membranes, sensors, electronics, composites, coatings
	Metal oxides (e.g., ZnO, TiO_2_)	Paints, coatings, adhesives, paper, plastics, rubber, printing inks, textiles, ceramics, floor coverings, roofing materials, water treatment agents, automotive products, catalysts
	Quantum dots (e.g., CdSe/ZnS)	Light emitting diodes, solar cells, photodetectors
FDA Center for Food Safety and Applied Nutrition	Boron nitride, nanocellulose, nanoclays, nanocopper, nanosilver, TiN	Food packaging
	TiO_2_, SiO_2_	Food packaging, direct food additive
CDC/NIOSH Health Effects Laboratory Division	Carbon-based nanomaterials: carbon nanotubes, carbon nanofiber, carbon black, graphene	Electronics, energy storage, automotive applications, structural engineering, pigments, sensors, medicine, etc.
	Complex mixtures containing nanometals and carbon in advanced manufacturing settings	3D printing
	ENM enabled composites including plastics and concrete, and coatings	Thermoplastics used for automotive parts, construction materials, optical and medical devices, circuitry, food and beverage packaging, high-pressure applications, paints and sealants, anti-corrosives, consumer products, etc.
	Metal and metal oxide nanoparticles (i.e., silver, TiO_2_, NiO, CuO, CeO_2_ with and without SiO_2_ coating, Fe_2_O_3_ with and without SiO_2_ coating)	Semiconductors, wafer polishing process called chemical mechanical planarization, mechanical glass polishing applications, electrical applications, cosmetics, proficient catalysts, medicine, disinfectants, imaging techniques, etc.
	Nanoclays	Plastic moldings, aircraft and automobile body cladding, thermoplastic, paints, waste treatments, etc.
	Non-carbon-based organics: i.e., nanocellulose	Food emulsions, biomedical applications including tissue replacements and drug delivery wood adhesives, water treatment, microbe and virus decontamination, air purification, etc.
USDA Forest Service Forest Products Laboratory	Nanocellulose	Paper, food packaging, lightweight automobile materials, concrete, zero-emission coatings, oil drilling, energy-efficient nanocellulose production, international standards development

a Represents emerging areas for EMN use as an antimicrobial pesticide.

CdSe, cadmium selenide; CeO_2_, cerium(IV) oxide; Fe_3_O_4_, iron (II,III) oxide; SiO_2_, silicon dioxide; TiN, titanium nitride, TiO_2_, titanium dioxide; ZnO, zinc oxide

**Tab. 2:** EPA test guidelines^6^ identified in the NanoWG survey that are relevant to ENM use cases

EPA guideline number	EPA guideline title	Substances/products tested	References
OCSPP 870.1100	Acute oral toxicity	Pesticides and pesticide formulations	EPA, 2002
OCSPP 870.1200	Acute dermal toxicity	Pesticides and pesticide formulations	EPA, 1998a
OCSPP 870.1300	Acute inhalation toxicity	Pesticides and pesticide formulations	EPA, 1998b
OCSPP 870.2400	Acute eye irritation	Pesticides and pesticide formulations	EPA, 1998c
OCSPP 870.2500	Acute dermal irritation	Pesticides and pesticide formulations	EPA, 1998d
OCSPP 870.2600	Skin sensitization	Pesticides and pesticide formulations	EPA, 2003
OCSPP 870.3050	Repeated dose 28-day oral toxicity study in rodents	Food additives and new dietary ingredients	EPA, 2000
OCSPP 870.3100	90-day oral toxicity in rodents	Food additives and new dietary ingredients, pesticides and pesticide formulations	EPA, 1998e
OCSPP 870.3250	Subchronic dermal toxicity 90 days	Pesticides and pesticide formulations	EPA, 1996a
OCSPP 870.3465	90-day inhalation toxicity	Pesticides and pesticide formulations	EPA, 1998f
OCSPP 870.3700	Prenatal developmental toxicity study	Pesticides and pesticide formulations	EPA, 1998g
OCSPP 870.3800	Reproduction and fertility effects	Pesticides and pesticide formulations	EPA, 1998h
OCSPP 870.4100	Chronic toxicity	Pesticides and pesticide formulations	EPA, 1998i
OCSPP 870.4200	Carcinogenicity	Pesticides and pesticide formulations	EPA, 1998j
OCSPP 870.5100	Bacterial reverse mutation test	Food additives and new dietary ingredients, pesticides and pesticide formulations	EPA, 1998k
OCSPP 870.5300	*In vitro* mammalian cell gene mutation test	Food additives and new dietary ingredients, pesticides and pesticide formulations	EPA, 1998l
OCSPP 870.5375	*In vitro* mammalian chromosome aberration test	Pesticides and pesticide formulations	EPA, 1996b
OCSPP 870.5385	*In vivo* mammalian cytogenetics tests: Bone marrow chromosomal analysis	Pesticides and pesticide formulations	EPA, 1998m
OCSPP 870.5395	*In vivo* mammalian cytogenetics tests: Erythrocyte micronucleus assay	Pesticides and pesticide formulations	EPA, 1998n
OCSPP 870.7485	Metabolism and pharmacokinetics	Pesticides and pesticide formulations	EPA, 1998o
OCSPP 870.7800	Immunotoxicity	Pesticides and pesticide formulations	EPA, 1998p

a In general, the responses focused on EPA guidelines most often used to evaluate risks to human health. This table should not be considered a complete compendium of all guidelines that may be used to evaluate the effects of ENMs.

**Tab. 3:** ENM guidance documents for industry

Agency	Guidance title	Products tested	References
CPSC	CPSC Nanomaterial Statement	Consumer products	CPSC, 2019
EPA	Working Guidance on EPA’s Section 8(a) Information Gathering Rule on Nanomaterials in Commerce	Chemicals/mixtures subject to TSCA regulation	EPA, 2017
FDA	Guidance for Industry: Considering Whether an FDA-Regulated Product Involves the Application of Nanotechnology	Products, materials, ingredients, and other substances regulated by FDA, including drugs, biological products, medical devices, food substances (including food for animals), dietary supplements, cosmetic products, and tobacco products	FDA, 2014b
	DRAFT Guidance for Industry: Drug Products, Including Biological Products, that Contain Nanomaterials	Human drug products, including those that are biological products, in which a nanomaterial (as explained in this section) is present in the finished dosage form; pharmaceuticals and biologics	FDA, 2017
	Guidance for Industry: Assessing the Effects of Significant Manufacturing Process Changes, Including Emerging Technologies, on the Safety and Regulatory Status of Food Ingredients and Food Contact Substances, Including Food Ingredients that are Color Additives	Food ingredients and food contact substances, including food ingredients that are color additives	FDA, 2014a
	Guidance for Industry – Safety of Nanomaterials in Cosmetic Products	Cosmetic products	FDA, 2014c
	Guidance for Industry: Use of Nanomaterials in Food for Animals	Animal feed	FDA, 2015
CDC/NIOSH	Approaches to Safe Nanotechnology: Managing the Health and Safety Concerns Associated with Engineered Nanomaterials	Engineered nanomaterials	CDC/NIOSH, 2009a
	Building a Safety Program to Protect the Nanotechnology Workforce: A Guide for Small to Medium-Sized Enterprises	Nanomaterials	CDC/NIOSH, 2016
	Controlling Health Hazards When Working with Nanomaterials: Questions to Ask Before You Start	Nanomaterials (a poster designed to guide workers on how to prevent exposures to nanomaterials)	CDC/NIOSH, 2018a
	Current Intelligence Bulletin 60: Interim Guidance for Medical Screening and Hazard Surveillance for Workers Potentially Exposed to Engineered Nanoparticles	Engineered nanomaterials	CDC/NIOSH, 2009b
CDC/NIOSH	Current Intelligence Bulletin 63: Occupational Exposure to Titanium Dioxide	Titanium dioxide	CDC/NIOSH, 2011
	Current Intelligence Bulletin 65: Occupational Exposure to Carbon Nanotubes and Nanofibers	Carbon nanotubes, nanofibers	CDC/NIOSH, 2013
	General Safe Practices for Working with Engineered Nanomaterials in Research Laboratories	Engineered nanomaterials (provides the best information currently available on engineering controls and safe work practices to be followed when working with ENMs in research laboratories, the front line of creating new nanomaterials, testing their usefulness and determining their toxicological and environmental impacts)	CDC/NIOSH, 2012
	Safe Nanotechnology in the Workplace	Nanoparticles (an introduction for employers, managers, and safety and health professionals)	CDC/NIOSH, 2008
	Workplace Design Solutions: Protecting Workers during Nanomaterial Reactor Operations	Nanomaterials (The controls described in this document include enclosures for large and small reactors during harvesting as well as an approach for controlling exposures during reactor cleaning.)	CDC/NIOSH, 2018c
	Workplace Design Solutions: Protecting Workers during the Handling of Nanomaterials	Nanomaterials (The controls described in this document include chemical fume hoods, nanomaterial handling enclosures, biological safety cabinets, and glove boxes.)	CDC/NIOSH, 2018d
	Workplace Design Solutions: Protecting Workers during Intermediate and Downstream Processing of Nanomaterials	Nanomaterials (The controls described in this document include local exhaust ventilation such as annular exhaust hoods, enclosures around the emission points, and downflow booths for larger scale processes.)	CDC/NIOSH, 2018b

**Tab. 4:** Potential measurements for dosimetry characterization of ENMs

ENM dosimetry measurement	Rationale for measurement	Potentially relevant analytical technique(s) and test methods^a^
ENM mass concentration in test media before exposure period	Determines the initial concentration; mass measurements are easier to measure than particle number or surface area concentrations.	Inductively coupled plasma-mass spectrometry (ICP-MS) (ASTM E3269-21, 2021)
ENM mass concentration in test media after exposure period	Determines the ENM concentration after exposure; mass measurements are easier to measure than particle number or surface area concentrations; the information at the beginning and end of the exposure period can enable determining the actual exposure concentration and changes in the ENM (e.g., dissolution) during the test.	ICP-MS^b^
ENM number or surface area concentration in test media before exposure period	Suggested to be more reflective of the toxicological risk than mass based ENM concentration, and thus better enable *in vitro* to *in vivo* extrapolation.	Single particle ICP-MS (spICP-MS), nanoparticle tracking analysis (NTA), transmission electron microscopy (TEM) (ISO, 2020a; ASTM E3269-21, 2021)
ENM number or surface area concentration in test media after exposure period	Suggested to be more reflective of the toxicological risk than mass based ENM concentration; testing before and after exposure period can reveal changes in the suspended ENMs such as agglomeration.	spICP-MS, NTA, TEM^c^
ENM mass concentration associated with cells after exposure period (if applicable)	Reveals information about the actual cellular exposure concentration; not applicable to *in chemico* methods.	ICP-MS (ASTM E3247-20, 2020; ASTM E3269-21, 2021)
Modeling of ENM mass concentration associated with cells after exposure period (if applicable)	Modeling the ENM cellular dose may better reflect the potential effects and could facilitate *in vivo* to *in vitro* extrapolation.	Modeling approaches include the ISDD and ISD3 models (DeLoid et al., 2017; Thomas et al., 2018)
ENM mean size prior to addition to test media	Provides fundamental information about the ENM to be tested and is broadly recommended.	Dynamic light scattering (DLS), spICP-MS, TEM, NTA (ISO, 2016, 2020a,b,c, 2021; ASTM E2834-12, 2018; ASTM E3247-20, 2020; ASTM E2490-09, 2021; ASTM WK68060, 2018)
ENM size distribution prior to addition to test media	Provides fundamental information about the ENM to be tested and is broadly recommended.	DLS, spICP-MS, TEM, NTA^d^
ENM mean size in test media prior to exposure period	Provides information about the ENM form (e.g., agglomerated or as individual particles) that is actually used in the test.	DLS, spICP-MS, TEM, NTA^d^
ENM mean size in test media after exposure period	Provides information about changes to the ENM form (e.g., agglomerated or as individual particles) during the test.	DLS, spICP-MS, TEM, NTA^d^
ENM size distribution in test media prior to exposure period	Provides information about the ENM form (e.g., agglomerated or as individual particles) that is actually used in the test.	DLS, spICP-MS, TEM, NTA^d^
ENM size distribution in test media after exposure period	Provides information about changes to the ENM form (e.g., agglomerated or as individual particles) during the test.	DLS, spICP-MS, TEM, NTA^d^
ENM dissolution in test media after exposure period	Provides information about changes to the ENM form during the test and may help with understanding the toxicity mechanism when compared to toxicity data from the dissolved form.	DLS, spICP-MS, TEM, NTA NTA^d^

a The techniques and test methods provided in this table may be potentially relevant but should not be considered the only potential methods that may be used, nor should they be considered relevant to all use cases.

b Citations are the same as those used for “ENM mass concentration in test media before exposure period”.

c Citations are the same as those used for “ENM number or surface area concentration in test media after exposure period”.

d Citations are the same as those used for “ENM size distribution prior to addition to test media”.

DLS, dynamic light scattering; ICP-MS, inductively-coupled plasma mass spectrometry; NTA, nanoparticle tracking analysis; spICP-MS, single particle ICP-MS; TEM, transmission electron microscopy

**Tab. 5:** Categories of *in vitro* test methods

Category	Exposure	Example of a standard method or guidance document
1	Liquid exposure to suspended molecules or suspended cells	*In chemico* skin sensitization: direct peptide reactivity assay (DPRA) (OECD, 2019)
2	Submerged liquid exposure with cells at the bottom of wells	*In vitro* skin sensitization: The ARE-Nrf2 luciferase KeratinoSens test method (OECD, 2018c)
3	A liquid, cream, or solid is directly applied to a biological test system such as a 3D construct	*In vitro* skin irritation: reconstructed human epidermis test method (OECD, 2021a)
4	Airborne exposure to a biological test system located on an air-liquid interface insert	Considerations for in vitro studies of airborne nano-objects and their aggregates and agglomerates (ISO, 2020d)
5	Exposure via multiple routes using an *in vitro* microphysiological system (e.g., eye-on-a-chip, gut-on-a-chip, and lung-on-a-chip devices)	Standard methods or guidance documents are not yet published to our knowledge.

**Tab. 6:** Summary of potential control experiments to identify assay artifacts^a^

Potential control experiments	Method to perform control experiment	Purpose(s)	Examples of relevant *in vitro* assays	References
Zero h control	Add the ENMs at a certain step of the assay and then immediately perform the remainder of the assay without modification; this differs from the typical approach in that there is no exposure period after the ENMs are added.	Test if ENMs: – Cause a toxicological effect (e.g., DNA damage) during processing steps after conclusion of exposure period by evaluating if effects could be observed during the processing steps after the assay is finished. – Would interact with test reagents or biomolecules and cause a false negative or false positive result. – May cause a change in the cell stability for suspended cells through heteroagglomeration.	Comet assay, DCFDA stress assay	Lin et al., 2009; Horst et al., 2013; Rösslein et al., 2013; Petersen et al., 2020
Cell free control	Add the ENMs only to the test media and perform the analytical method, or add the ENMs with the assay’s reagents and then perform the assay.	Assess if ENMs themselves, in the absence of cells, produce or inhibit a signal (e.g., absorbance, fluorescence) or interact with assay reagents in a way that could produce or inhibit the production of a signal similar to the assay measurement. This will identify interferences and potential false positive (or false negative) results.	All absorbance and fluorescence-based assays; DCFDA assay	Wörle-Knirsch et al., 2006; Horst et al., 2013; Petersen et al., 2014; Elliott et al., 2017
Nutrient depletion control	Incubate ENM with assay medium for the duration of the assay, remove ENMs such as by using filtration, and perform assay with the medium.	Assess the extent to which adsorption of media constituents by ENMs could have an indirect toxicity effect on endpoints.	All assays	Chang et al., 2011; Zhao et al., 2013; Petersen et al., 2014
Positive spiked control (inhibition/enhancement control)	Perform the assay exposure period with the positive control. Then, add the ENMs to the positive control wells and perform subsequent analysis steps.	Assess if the presence of ENMs may inhibit/enhance the signal of cells that would otherwise have a positive response in the assay.	Flow cytometry assays, absorbance, and fluorescence assays	Keene et al., 2014; Bohmer et al., 2018

a This table has been modified and edited with permission from Petersen et al. (2014), © 2014 American Chemical Society.

DCFDA, 2’, 7’-dichlorodihydrofluorescein diacetate

**Tab. 7:** Potential control experiments to understand toxicity mechanisms and support interpretation of assay results^a^

Potential control experiments	Method to perform control experiment	Purpose(s)	References
Coating control	Perform the assay using the ENM coating at a relevant coating concentration.	Test if coating has toxicological or biological effects on organisms or cells.	Petersen et al., 2011; Sun et al., 2017
Dispersant control	Perform the assay using the ENM dispersant at a relevant dispersant concentration.	Test if coating has toxicological or biological effects on organisms or cells.	Wang et al., 2010; Youn et al., 2012
Dissolved ion control	For ENMs that dissolve, perform the assay using the dissolved ion.	Allows for comparison of endpoints between. ENM and constituent dissolved ions. Assess if ENM formation could occur from ions in test media or in cells present during the assay	Scanlan et al., 2013
Filtrate only control	Filter the ENM suspension and then perform assay with the filtrate.	Assess potential toxicity of contaminants, and dissolution from ENMs during the synthesis, storage, and dispersion processes	Hanna et al., 2016; Coyle et al., 2020

a This table has been modified and edited with permission from Petersen et al. (2014), © 2014 American Chemical Society.

## Footnotes

1
http://nanodb.dk/

2
https://ntp.niehs.nih.gov/go/natl-strategy

3 https://ntp.niehs.nih.gov/pubhealth/evalatm/iccvam/iccvam-agencies/index.html (accessed 07.08.2019)

4
https://ntp.niehs.nih.gov/iccvamreport/2019/about/index.html

5
https://ntp.niehs.nih.gov/go/iccvam-wg

6
https://www.oecd.org/chemicalsafety/testing/series-testing-assessment-publications-number.htm

7
https://www.epa.gov/reviewing-new-chemicals-under-toxic-substances-control-act-tsca/fact-sheet-nanoscale-materials

8 This document is a draft and not for implementation. Once finalized, the document will represent the FDA’s position.

9
https://www.cdc.gov/niosh/topics/nanotech/default.html

10 doi:10.14573/altex.2105041s

## Abbreviations

AOP: adverse outcome pathway
ASTM: American Society for Testing and Materials International
CDC/NIOSH: Centers for Disease Control and Prevention/National Institute for Occupational Safety and Health
CFSAN: FDA Center for Food Safety and Applied Nutrition
CPSC: U.S. Consumer Product Safety Commission
ENM: engineered nanomaterials
EPA: U.S. Environmental Protection Agency
FDA: U.S. Food and Drug Administration
FIFRA: Federal Insecticide, Fungicide, and Rodenticide Act
FQPA: Food Quality Protection Act
ICCVAM: Interagency Coordinating Committee on the Validation of Alternative Methods
ISO: International Organization for Standardization
NAMs: new approach methodologies
NanoWG: ICCVAM Nanomaterials Workgroup
NIST: National Institute of Standards and Technology
NTRC: Nanotechnology Research Center
OECD: Organisation for Economic Co-operation and Development
OPP: EPA Office of Pesticide Programs
OPPT: EPA Office of Pollution Prevention and Toxics
TSCA: Toxic Substances Control Act
