# Supplementary material for: U.S. Federal Agency Interests and Key Considerations for New Approach Methodologies for Nanomaterials
Source: ALTEX. Author manuscript; Available in PMC 2022 May 18. (PMC9115850; doi:10.14573/altex.2105041)
Supplement: BD5FA2B27A44129C49353F68BEC5AEB6 [file NIHMS1792464-supplement-BD5FA2B27A44129C49353F68BEC5AEB6.pdf]

Petersen et al.:

U.S. Federal Agency Interests and Key Considerations for New Approach Methodologies for Nanomaterials

Supplementary Data

Tab. S1: Selected ASTM, ISO, and OECD nanomaterials guidances[a]

Agency or documentary standards organizations	Status	Document number	Title	Reference
ASTM	Draft under development	WK60553	New Guide for Evaluation of Nanoparticulate Material Internalization by Phagocytic Cells *In Vitro*	ASTM WK60553, 2017
		WK60554	New Test Method for Detection of Nitric Oxide Production	ASTM WK60554, 2017
	In balloting	WK63310	New Guide for Visualization and Identification of Nanomaterials in Biological and Nonbiological Matrices Using Darkfield Microscopy with Hyperspectral Imaging Analysis	ASTM WK63310, 2018
	Published/active	ASTM E2524-08	Standard Test Method for Analysis of Hemolytic Properties of Nanoparticles	ASTM E2524-08, 2013
		ASTM E2525-08	Standard Test Method for Evaluation of the Effect of Nanoparticulate Materials on the Formation of Mouse Granulocyte-Macrophage Colonies	ASTM E2525-08, 2013
		ASTM E2526-08	Standard Test Method for Evaluation of Cytotoxicity of Nanoparticulate Materials in Porcine Kidney Cells and Human Hepatocarcinoma Cells	ASTM E2525-08, 2013
		ASTM E3238-20	Standard Test Method for Quantitative Measurement of the Chemoattractant Capacity of a Nanoparticulate Material *In Vitro*	ASTM E3238-20, 2020
ISO	Under development	ISO/CD TS 19337	Nanotechnologies – Characteristics of Working Suspensions of Nano-Objects for *In Vitro* Assays to Evaluate Inherent Nano-Object Toxicity	ISO/CD TS 19337, not yet available
		ISO/DTR 22455	Nanotechnologies – High Throughput Screening Method for Nanoparticles Toxicity using 3D Cells	ISO/DTR 22455, not yet available
		ISO/DTR 23463	Nanotechnologies – Characterization of Carbon Nanotube and Carbon Nanofiber Aerosols in Relation to Inhalation Toxicity Tests	ISO/DTR 23463, not yet available
		ISO/DTS 21357	Nanotechnologies – Evaluation of the Mean Size of Nano-Objects in Liquid Dispersions by Static Multiple Light Scattering (SMLS)	ISO/DTS 21357, not yet available
		ISO/DTS 23302	Nanotechnologies – Guidance on Measurands for Characterising Nano-Objects and Materials that Contain Them	ISO/DTS 23302, not yet available
		ISO/PRF TS 21633	Nanotechnologies – Label-Free Impedance Technology to Assess the Toxicity of Nanomaterials *In Vitro*	ISO/PRF TS 21633, not yet available
		ISO/PRF TS 23034	Nanotechnologies – Method to Estimate Cellular Uptake of Carbon Nanomaterials using Optical Absorption	ISO/PRF TS 23034, not yet available
		ISO/WD TR 24672	Nanotechnologies – Guidance on the Measurement of Nanoparticle Number Concentration	ISO/WD TR 24672, not yet available
		ISO/WD TR 5387	Nanotechnologies – Lung Burden Measurement of Nanomaterials for Inhalation Toxicity Studies	ISO/WD TR 5387, not yet available

doi:10.14573/altex.2105041s

This is an Open Access article distributed under the terms of the Creative Commons Attribution 4.0 International license (http://creativecommons.org/licenses/by/4.0/), which permits unrestricted use, distribution and reproduction in any medium, provided the original work is appropriately cited.

Agency or documentary standards organizations	Status	Document number	Title	Reference
ISO	Under development	ISO/WD TS 5094	Nanotechnologies – Assessment of Peroxidase-Like Activity of Metal and Metal Oxide Nanoparticles	ISO/WD TS 5094, not yet available
ISO	Published/active	ISO 10801:2010	Nanotechnologies – Generation of Metal Nanoparticles for Inhalation Toxicity Testing using the Evaporation/Condensation Method	ISO, 2010a
		ISO 10808:2010	Nanotechnologies – Characterization of Nanoparticles in Inhalation Exposure Chambers for Inhalation Toxicity Testing	ISO, 2010b
		ISO 19007:2018	Nanotechnologies – *In Vitro* MTS Assay for Measuring the Cytotoxic Effect of Nanoparticles	ISO, 2018a
		ISO 20814:2019	Nanotechnologies – Testing the Photocatalytic Activity of Nanoparticles for NADH Oxidation	ISO, 2019a
		ISO/29701:2010	Nanotechnologies – Endotoxin Test on Nanomaterial Samples for *In Vitro* Systems – *Limulus* Amebocyte Lysate (LAL) Test	ISO, 2010c
		ISO/TR 13014:2012	Nanotechnologies – Guidance on Physicochemical Characterization of Engineered Nanoscale Materials for Toxicologic Assessment	ISO, 2012a
		ISO/TR 13121:2011	Nanotechnologies – Nanomaterial Risk Evaluation	ISO, 2011
ISO	Published/active	ISO/TR 16196:2016	Nanotechnologies – Compilation and Description of Sample Preparation and Dosing Methods for Engineered and Manufactured Nanomaterials	ISO, 2016a
		ISO/TR 16197:2014	Nanotechnologies – Compilation and Description of Toxicological Screening Methods for Manufactured Nanomaterials	ISO, 2014a
		ISO/TR 18637:2016	Nanotechnologies – Overview of Available Frameworks for the Development of Occupational Exposure Limits and Bands for Nano-Objects and their Aggregates and Agglomerates (NOAAs)	ISO, 2016b
		ISO/TR 19057:2017	Nanotechnologies – Use and Application of Acellular *In Vitr*o Tests and Methodologies to Assess Nanomaterial Biodurability	ISO, 2017a
		ISO/TR 19601:2017	Nanotechnologies – Aerosol Generation for Air Exposure Studies of Nano-Objects and their Aggregates and Agglomerates	ISO, 2017b
		ISO/TR 21624:2020	Nanotechnologies – Considerations for *In Vitro* Studies of Airborne Nano-Objects and their Aggregates and Agglomerates (NOAA)	ISO, 2020a
		ISO/TR 22019:2019	Nanotechnologies – Considerations for Performing Toxicokinetic Studies with Nanomaterials	ISO, 2019b
		ISO/TS 12901-1:2012	Nanotechnologies – Occupational Risk Management Applied to Engineered Nanomaterials Part 1: Principles and Approaches	ISO, 2012b
		ISO/TS 12901-2:2014	Nanotechnologies – Occupational Risk Management Applied to Engineered Nanomaterials – Part 2: Use of the Control Banding Approach	ISO, 2014b
ISO	Published/active	ISO/TS 16195:2013	Nanotechnologies – Generic Requirements for Reference Materials for Development of Methods for Characteristic Testing, Performance Testing and Safety Testing of Nano-Particle and Nano-Fiber Powders [Replaces ISO/TS 16195 (2013)]	ISO, 2018b
		ISO/TS 16550:2014	Nanotechnologies – Determination of Silver Nanoparticles Potency by Release of Muramic Acid from *Staphylococcus aureus*	ISO, 2014c
		ISO/TS 18827:2017	Nanotechnologies – Electron Spin Resonance (ESR) as a Method for Measuring Reactive Oxygen Species (ROS) Generated by Metal Oxide Nanomaterials	ISO, 2017c
		ISO/TS 19006:2016	Nanotechnologies – 5-(and 6)-Chloromethyl-2’,7’ dichloro-dihydrofluorescein diacetate (CM-H2DCF-DA) Assay for Evaluating Nanoparticle-Induced Intracellular Reactive Oxygen Species (ROS) Production in RAW 264.7 Macrophage Cell Line	ISO, 2016c

Agency or documentary standards organizations	Status	Document number	Title	Reference
		ISO/TS 19337:2016	Nanotechnologies – Characteristics of Working Suspensions of Nano-Objects for *In Vitro* Assays to Evaluate Inherent Nano-Object Toxicity	ISO, 2016d
		ISO/TS 20660:2019	Nanotechnologies – Antibacterial Silver Nanoparticles – Specification of Characteristics and Measurement Methods	ISO, 2019c
		ISO/TS 20787:2017	Nanotechnologies – Aquatic Toxicity Assessment of Nanomaterials in Saltwater Lakes using *Artemia sp*. Nauplii	ISO, 2017d
		ISO/TS 21236-1:2019	Nanotechnologies – Clay Nanomaterials – Part 1: Specification of Characteristics and Measurement Methods for Layered Clay Nanomaterials	ISO, 2019d
		ISO/TS 22082:2020	Nanotechnologies – Assessment of Nanomaterial Toxicity using Dechorionated Zebrafish Embryo	ISO, 2020b
		ISO/TS 23459:2021	Nanotechnologies – Assessment of Protein Secondary Structure during an Interaction with Nanomaterials using Ultraviolet Circular Dichroism	ISO, 2021
OECD	Published/active	GD 39	Guidance Document on Inhalation Toxicity Studies	OECD, 2009a
		GD 317	Guidance Document on Aquatic and Sediment Toxicological Testing of Nanomaterials	OECD, 2020a
		TG 318	Guidance Document for the Testing of Dissolution and Dispersion Stability of Nanomaterials and the Use of the Data for Further Environmental Testing and Assessment Strategies	OECD, 2017b
		TG 412	Subacute Inhalation Toxicity: 28-Day Study	OECD, 2018c
		TG 413	Subchronic Inhalation Toxicity: 90-Day Study	OECD, 2018d
		TG 433	Acute Inhalation Toxicity - Fixed Concentration Procedure	OECD, 2018e
OECD	Published/active	ENV/JM/MONO (2009)21	Preliminary Review of OECD Test Guidelines for their Applicability to Manufactured Nanomaterials	OECD, 2009b
		ENV/JM/MONO (2009)20/REV	Guidance Manual for the Testing of Manufactured Nanomaterials: OECD's Sponsorship Programme: First Revision	OECD, 2010a
		ENV/JM/MONO (2010)46	List of Manufactured Nanomaterials and List of Endpoints for Phase One of the Sponsorship Programme for the Testing of Manufactured Nanomaterials: Revision	OECD, 2010b
		ENV/JM/MONO (2012)40	Guidance on Sample Preparation and Dosimetry for the Safety Testing of Nanomaterials	OECD, 2012
		ENV/JM/MONO (2016)63	Alternative Testing Strategies in Risk Assessment of Manufactured Nanomaterials: Current State of Knowledge and Research Needs to Advance Their Use.	OECD, 2017a
		ENV/JM/MONO (2018)4	Evaluation of *In Vitro* Methods for Human Hazard Assessment Applied in the OCED Testing Programme for the Safety of Manufactured Nanomaterials	OECD, 2018a
		ENV/JM/MONO (2018)24	Investigating the Different Types of Risk Assessments of Manufactured Nanomaterials. Identifying Tools Available for Risk Management Measures and Uncertainties Driving Nano-Specific Data Needs	OECD, 2018b
		ENV/JM/MONO (2019)12	Physical-chemical Decision Framework to Inform Decisions for Risk Assessment of Manufactured Nanomaterials	OECD, 2019a
		ENV/JM/MONO (2019)13	Guiding Principles for Measurements and Reporting for Nanomaterials: Physical Chemical Properties	OECD, 2019b
		ENV/JM/MONO (2020)32	Ability of Biopersistent/Biodurable Manufactured Nanomaterials (MNs) to Induce Lysosomal Membrane Permeabilization (LMP) as a Prediction of Their Long-Term Toxic Effects	OECD, 2020b

Agency or documentary standards organizations	Status	Document number	Title	Reference
		ENV/JM/MONO (2020)33	Advancing Adverse Outcome Pathway (AOP) Development for Nanomaterial Risk Assessment and Categorisation Part 1: Final Project Report and Recommendations with Methodology to Prioritise Key Events (KEs) Relevant for Manufactured Nanomaterials	OECD, 2020c
		ENV/JM/MONO (2020)34	Advancing Adverse Outcome Pathway (AOP) Development for Nanomaterial Risk Assessment and Categorisation Part 2: Case Study on Tissue Injury	OECD, 2020d
		ENV/JM/MONO (2020)35	Advancing Adverse Outcome Pathway (AOP) Development for Nanomaterial Risk Assessment and Categorisation Part 3: Workshop Report and Recommendations	OECD, 2020e

[a]This table is not intended to be a complete compendium of ASTM, ISO, or OECD documents related to nanomaterials.
Additional relevant documents may be found at:

- https://www.astm.org/Standards/nanotechnology-standards.html
- https://www.iso.org/standards.html
- https://www.oecd.org/env/ehs/testing/oecdguidelinesforthetestingofchemicals.htm
- http://www.oecd.org/env/ehs/nanosafety/publications-series-safety-manufactured-nanomaterials.htm

References

ASTM E2524-08 (2013). Standard Test Method for Analysis of Hemolytic Properties of Nanoparticles. West Conshohocken, PA: ASTM International. doi:10.1520/E2524-08R13

ASTM E2525-08 (2013). Test Method for Evaluation of the Effect of Nanoparticulate Materials on the Formation of Mouse Granulocyte-Macrophage Colonies. ASTM International. doi:10.1520/E2525-08R13

ASTM E3238-20 (2020). Standard Test Method for Quantitative Measurement of the Chemoattractant Capacity of a Nanoparticulate Material In Vitro. West Conshohocken, PA: ASTM International. doi:10.1520/E3238-20

ASTM WK60553 (2017). New Guide for Evaluation of Nanoparticulate Material Internalization by Phagocytic Cells In Vitro. West Conshohocken, PA: ASTM International. https://www.astm.org/DATABASE.CART/WORKITEMS/WK60553.htm (accessed 21.10.2020).

ASTM WK60554 (2017). New Test Method for Detection of Nitric Oxide Production. West Conshohocken, PA: ASTM International. https://www.astm.org/DATABASE.CART/WORKITEMS/WK60554.htm (accessed 21.10.2020).

ASTM WK63310 (2018). New Guide for Visualization and Identification of Nanomaterials in Biological and Nonbiological Matrices Using Darkfield Microscopy with Hyperspectral Imaging Analysis. West Conshohocken, PA: ASTM International. https://www.astm.org/DATABASE.CART/WORKITEMS/WK63310.htm (accessed 21.10.2020).

ISO (2010a). ISO 10801:2010 Nanotechnologies – Generation of Metal Nanoparticles for Inhalation Toxicity Testing using the Evaporation/Condensation Method. https://www.iso.org/cms/render/live/en/sites/isoorg/contents/data/standard/04/61/46129.html (accessed 21.10.2020).

ISO (2010b). ISO 10808:2020 Nanotechnologies – Characterization of Nanoparticles in Inhalation Exposure Chambers for Inhalation Toxicity Testing. https://www.iso.org/cms/render/live/en/sites/isoorg/contents/data/standard/04/61/46130.html (accessed 21.10.2020).

ISO (2010c). ISO 29701:2010 Nanotechnologies – Endotoxin Test on Nanomaterial Samples for In Vitro Systems – Limulus amebocyte Lysate (LAL) Test. https://www.iso.org/cms/render/live/en/sites/isoorg/contents/data/standard/04/56/45640.html (accessed 22.10.2020).

ISO (2011). ISO/TR 13121:2011 Nanotechnologies – Nanomaterial Risk Evaluation. https://www.iso.org/cms/render/live/en/sites/isoorg/contents/data/standard/05/29/52976.html (accessed 22.10.2020).

ISO (2012a). ISO/TR 13014:2012 Nanotechnologies – Guidance on Physicochemical Characterization of Engineered Nanoscale Materials for Toxicologic Assessment. https://www.iso.org/cms/render/live/en/sites/isoorg/contents/data/standard/05/23/52334.html (accessed 22.10.2020).

ISO (2012b). ISO/TS 12901-1:2012 Nanotechnologies – Occupational Risk Management Applied to Engineered Nanomaterials Part 1: Principles and Approaches.

https://www.iso.org/cms/render/live/en/sites/isoorg/contents/data/standard/05/21/52125.html (accessed 22.10.2020).
ISO (2013). ISO/TS 16195:2013 – Nanotechnologies – Generic Requirements for Reference Materials for Development of Methods for Characteristic Testing, Performance Testing and Safety Testing of Nano-Particle and Nano-Fiber Powders. https://webstore.ansi.org/Standards/ISO/ISOTS161952013 (accessed 22.10.2020).
ISO (2014a). ISO/TR 16197:2014 Nanotechnologies – Compilation and Description of Toxicological Screening Methods for Manufactured Nanomaterials. https://www.iso.org/cms/render/live/en/sites/isoorg/contents/data/standard/05/58/55827.html (accessed 22.10.2020).
ISO (2014b). ISO/TS 12901-2:2014 Nanotechnologies – Occupational Risk Management Applied to Engineered Nanomaterials – Part 2: Use of the Control Banding Approach. https://www.iso.org/cms/render/live/en/sites/isoorg/contents/data/standard/05/33/53375.html (accessed 22.10.2020).
ISO (2014c). ISO/TS 16550:2014 Nanotechnologies – Determination of Silver Nanoparticles Potency by Release of Muramic Acid from Staphylococcus aureus. https://www.iso.org/cms/render/live/en/sites/isoorg/contents/data/standard/05/70/57084.html (accessed 22.10.2020).
ISO (2016a). ISO/TR 16196:2016 Nanotechnologies – Compilation and Description of Sample Preparation and Dosing Methods for Engineered and Manufactured Nanomaterials. https://www.iso.org/cms/render/live/en/sites/isoorg/contents/data/standard/05/58/55826.html (accessed 22.10.2020).
ISO (2016b). ISO/TR 18637:2016 Nanotechnologies – Overview of Available Frameworks for the Development of Occupational Exposure Limits and Bands for Nano-Objects and their Aggregates and Agglomerates (NOAAs). https://www.iso.org/cms/render/live/en/sites/isoorg/contents/data/standard/06/30/63096.html (accessed 22.10.2020).
ISO (2016c). ISO/TS 19006:2016 Nanotechnologies – 5-(and 6)-Chloromethyl-2',7' dichloro-dihydrofluorescein diacetate (CM-H2DCF-DA) Assay for Evaluating Nanoparticle-Induced Intracellular Reactive Oxygen Species (ROS) Production in RAW 264.7 Macrophage Cell Line. https://www.iso.org/cms/render/live/en/sites/isoorg/contents/data/standard/06/36/63697.html (accessed 22.10.2020).
ISO (2016d). ISO/TS 19337:2016 Nanotechnologies – Characteristics of Working Suspensions of Nano-Objects for *In Vitro* Assays to Evaluate Inherent Nano-Object Toxicity. https://www.iso.org/cms/render/live/en/sites/isoorg/contents/data/standard/06/46/64652.html (accessed 22.10.2020).
ISO (2017a). ISO/TR 19057:2017 Nanotechnologies – Use and Application of Acellular In Vitro Tests and Methodologies to Assess Nanomaterial Biodurability. https://www.iso.org/cms/render/live/en/sites/isoorg/contents/data/standard/06/38/63836.html (accessed 22.10.2020).
ISO (2017b). ISO/TR 19601:2017 Nanotechnologies – Aerosol generation for air exposure studies of nano-objects and their aggregates and agglomerates (NOAA). https://www.iso.org/cms/render/live/en/sites/isoorg/contents/data/standard/06/54/65451.html (accessed 22.10.2020).
ISO (2017c). ISO/TS 18827:2017 Electron Spin Resonance (ESR) as a Method for Measuring Reactive Oxygen Species (ROS) Generated by Metal Oxide Nanomaterials. https://www.iso.org/cms/render/live/en/sites/isoorg/contents/data/standard/06/35/63502.html (accessed 22.10.2020).
ISO (2017d). ISO/TS 20787:2017 Nanotechnologies – Aquatic Toxicity Assessment of Nanomaterials in Saltwater Lakes using Artemia sp. Nauplii. https://www.iso.org/cms/render/live/en/sites/isoorg/contents/data/standard/06/90/69087.html (accessed 22.10.2020).
ISO (2018a). ISO 19007:2018 Nanotechnologies – In Vitro MTS Assay for Measuring the Cytotoxic Effect of Nanoparticles. https://www.iso.org/cms/render/live/en/sites/isoorg/contents/data/standard/06/36/63698.html (accessed 22.10.2020).
ISO (2018b). ISO/TS 16195:2018 Nanotechnologies – Specification for developing representative test materials consisting of nano-objects in dry powder form. https://www.iso.org/cms/render/live/en/sites/isoorg/contents/data/standard/07/31/73130.html (accessed 22.10.2020).
ISO (2019a). ISO 20814:2019 Nanotechnologies – Testing the Photocatalytic Activity of Nanoparticles for NADH Oxidation. https://www.iso.org/standard/69298.html
ISO (2019b). ISO/TR 22019:2019 Nanotechnologies – Considerations for Performing Toxicokinetic Studies with Nanomaterials. https://www.iso.org/cms/render/live/en/sites/isoorg/contents/data/standard/07/23/72381.html (accessed 22.10.2020).

ISO (2019c). ISO/TS 20660:2019 Nanotechnologies – Antibacterial Silver Nanoparticles – Specification of Characteristics and Measurement Methods. https://www.iso.org/cms/render/live/en/sites/isoorg/contents/data/standard/06/87/68771.html (accessed 22.10.2020).
ISO (2019d). ISO/TS 21236-1:2019 Nanotechnologies – Clay Nanomaterials – Part 1: Specification of Characteristics and Measurement Methods for Layered Clay Nanomaterials. https://www.iso.org/standard/70216.html?browse=tc
ISO (2020a). ISO/TR 21624:2020 Nanotechnologies – Considerations for In Vitro Studies of Airborne Nano-Objects and their Aggregates and Agglomerates (NOAA). https://www.iso.org/cms/render/live/en/sites/isoorg/contents/data/standard/07/12/71273.html (accessed June 4, 2020).
ISO (2020b). ISO/TS 22082:2020 Nanotechnologies – Assessment of Nanomaterial Toxicity using Dechorionated Zebrafish Embryo. https://www.iso.org/cms/render/live/en/sites/isoorg/contents/data/standard/07/25/72516.html (accessed 22.10.2020).
ISO (2021). ISO/TS 23459:2021 Nanotechnologies – Assessment of Protein Secondary Structure during an Interaction with Nanomaterials using Ultraviolet Circular Dichroism. https://www.iso.org/cms/render/live/en/sites/isoorg/contents/data/standard/07/56/75638.html (accessed 22.10.2020).
ISO/CD TS 19337 (not yet available). ISO/CD TS 19337 Nanotechnologies – Characteristics of Working Suspensions of Nano-Objects for In Vitro Assays to Evaluate Inherent Nano-Object Toxicity. https://www.iso.org/cms/render/live/en/sites/isoorg/contents/data/standard/08/05/80583.html (accessed April 15, 2021a).
ISO/DTR 22455 (not yet available). ISO/DTR 22455 Nanotechnologies – High Throughput Screening Method for Nanoparticles Toxicity using 3D Cells. https://www.iso.org/cms/render/live/en/sites/isoorg/contents/data/standard/07/32/73244.html (accessed October 22, 2020b).
ISO/DTR 23463 (not yet available). ISO/DTR 23463 Nanotechnologies – Characterization of Carbon Nanotube and Carbon Nanofiber Aerosols in Relation to Inhalation Toxicity Tests. https://www.iso.org/cms/render/live/en/sites/isoorg/contents/data/standard/07/56/75639.html (accessed October 22, 2020c).
ISO/DTS 21357 (not yet available). ISO/DTS 21357 Nanotechnologies – Evaluation of the Mean Size of Nano-Objects in Liquid Dispersions by Static Multiple Light Scattering (SMLS). https://www.iso.org/cms/render/live/en/sites/isoorg/contents/data/standard/07/07/70759.html (accessed April 15, 2021d).
ISO/DTS 23302 (not yet available). ISO/DTS 23302 Nanotechnologies – Guidance on Measurands for Characterising Nano-Objects and Materials that Contain Them. https://www.iso.org/cms/render/live/en/sites/isoorg/contents/data/standard/07/51/75190.html (accessed April 15, 2021e).
ISO/PRF TS 21633 (not yet available). ISO/PRF TS 21633 Nanomaterials – Label-Free Impedance Technology to Assess the Toxicity of Nanomaterials In Vitro. https://www.iso.org/cms/render/live/en/sites/isoorg/contents/data/standard/07/12/71295.html (accessed October 22, 2020f).
ISO/PRF TS 23034 (not yet available). ISO/PRF TS 23034 Nanotechnologies – Method to Estimate Cellular Uptake of Carbon Nanomaterials using Optical Absorption. https://www.iso.org/cms/render/live/en/sites/isoorg/contents/data/standard/07/43/74368.html (accessed October 22, 2020g).
ISO/WD TR 5387 (not yet available). ISO/WD TR 5387 Nanotechnologies – Lung Burden Measurement of Nanomaterials for Inhalation Toxicity Studies. https://www.iso.org/cms/render/live/en/sites/isoorg/contents/data/standard/08/12/81226.html (accessed April 15, 2021h).
ISO/WD TR 24672 (not yet available). ISO/WD TR 24672 Nanotechnologies – Guidance on the Measurement of Nanoparticle Number Concentration. https://www.iso.org/cms/render/live/en/sites/isoorg/contents/data/standard/07/93/79369.html (accessed April 15, 2021i).
ISO/WD TS 5094 (not yet available). ISO/WD TS 5094 Nanotechnologies – Assessment of Peroxidase-Like Activity of Metal and Metal Oxide Nanoparticles. https://www.iso.org/cms/render/live/en/sites/isoorg/contents/data/standard/08/07/80771.html (accessed 15.04.2021j).
OECD (2009a). Guidance Document on Inhalation Toxicity Studies. *OECD Series on Testing and Assessment No. 39*. OECD Publishing, Paris. https://www.oecd.org/officialdocuments/publicdisplaydocumentpdf/?cote=env/jm/mono(2009)28/rev1&doclanguage=en (accessed 23.10.2020).

OECD (2009b). Preliminary Review of OECD Test Guidelines for their Applicability to Manufactured Nanomaterials. http://www.oecd.org/officialdocuments/publicdisplaydocumentpdf/?doclanguage=en&cote=env/jm/mono(2009)21 (accessed 27.08.2020).

OECD (2010a). Guidance Manual for the Testing of Manufactured Nanomaterials: OECD's Sponsorship Programme: First Revision. *OECD Series on the Safety of Manufactured Nanomaterials No. 25*. OECD Publishing, Paris. http://www.oecd.org/officialdocuments/publicdisplaydocumentpdf/?cote=env/jm/mono(2009)20/rev&doclanguage=en

OECD (2010b). List of Manufactured Nanomaterials and List of Endpoints for Phase One of the Sponsorship Programme for the Testing of Manufactured Nanomaterials: Revision. *OECD Series on the Safety of Manufactured Nanomaterials No. 27*. OECD Publishing, Paris. http://www.oecd.org/officialdocuments/publicdisplaydocumentpdf/?cote=env/jm/mono(2010)46&doclanguage=en

OECD (2012). Guidance on Sample Preparation and Dosimetry for the Safety Testing of Nanomaterials. OECD Publishing, Paris. *OECD Series on the Safety of Manufactured Nanomaterials No. 36*. http://whttp://www.oecd.org/officialdocuments/publicdisplaydocumentpdf/?cote=env/jm/mono(2012)40&doclanguage=enww.oecd.org/officialdocuments/publicdisplaydocumentpdf/?cote=env/jm/mono(2009)20/rev&doclanguage=en

OECD (2017a). Alternative Testing Strategies in Risk Assessment of Manufactured Nanomaterials: Current State of Knowledge and Research Needs to Advance Their Use. *OECD Series on the Safety of Manufactured Nanomaterials No. 80.* OECD Publishing, Paris. http://www.oecd.org/officialdocuments/publicdisplaydocumentpdf/?cote=ENV/JM/MONO(2016)63&doclanguage=en

OECD (2017b). Test No. 318: Dispersion Stability of Nanomaterials in Simulated Environmental Media. OECD Publishing, Paris. https://www.oecd-ilibrary.org/environment/test-no-318-dispersion-stability-of-nanomaterials-in-simulated-environmental-media_9789264284142-en (accessed October 23, 2020).

OECD (2018a). Evaluation of In Vitro Methods for Human Hazard Assessment Applied in the OCED Testing Programme for the Safety of Manufactured Nanomaterials. *OECD Series on the Safety of Manufactured Nanomaterials No. 85.* OECD Publishing, Paris. http://www.oecd.org/officialdocuments/publicdisplaydocumentpdf/?cote=ENV/JM/MONO(2018)4&doclanguage=en

OECD (2018b). Investigating the Different Types of Risk Assessments of Manufactured Nanomaterials. Identifying Tools Available for Risk Management Measures and Uncertainties Driving Nano-Specific Data Needs. *OECD Series on the Safety of Manufactured Nanomaterials No. 88.* OECD Publishing, Paris. https://www.oecd.org/officialdocuments/publicdisplaydocumentpdf/?cote=env/jm/mono(2018)24&doclanguage=en

OECD (2018c). Test No. 412: Subacute Inhalation Toxicity: 28-Day Study. OECD Publishing, Paris. https://read.oecd-ilibrary.org/environment/test-no-412-subacute-inhalation-toxicity-28-day-study_9789264070783-en (accessed October 23, 2020).

OECD (2018d). Test No. 413: Subchronic Inhalation Toxicity: 90-day Study. OECD Publishing, Paris. https://www.oecd.org/env/test-no-413-subchronic-inhalation-toxicity-90-day-study-9789264070806-en.htm

OECD (2018e). Test No. 433: Acute Inhalation Toxicity: Fixed Concentration Procedure. OECD Publishing, Paris. https://www.oecd-ilibrary.org/environment/test-no-433-acute-inhalation-toxicity-fixed-concentration-procedure_9789264284166-en (accessed October 23, 2020).

OECD (2019a). Physical-chemical Decision Framework to Inform Decisions for Risk Assessment of Manufactured Nanomaterials. *OECD Series on the Safety of Manufactured Nanomaterials No. 90.* OECD Publishing, Paris. http://www.oecd.org/officialdocuments/publicdisplaydocumentpdf/?cote=env/jm/mono(2019)12&doclanguage=en

OECD (2019b). Guiding Principles for Measurements and Reporting for Nanomaterials: Physical Chemical Properties. *OECD Series on the Safety of Manufactured Nanomaterials No. 91.* OECD Publishing, Paris. http://www.oecd.org/officialdocuments/publicdisplaydocumentpdf/?cote=env/jm/mono(2019)13&doclanguage=en

OECD (2020a). Guidance Document on Aquatic and Sediment Toxicity Testing of Nanomaterials. *OECD Series on Testing and Assessment No. 317.* OECD Publishing, Paris. http://www.oecd.org/officialdocuments/publicdisplaydocumentpdf/?cote=env/jm/mono(2020)8&doclanguage=en (accessed October 23, 2020).

OECD (2020b). Ability of Biopersistent/Biodurable Manufactured Nanomaterials (MNs) to Induce Lysosomal Membrane Permeabilization (LMP) as a Prediction of Their Long-Term Toxic Effects. *OECD Series on the Safety of Manufactured Nanomaterials No. 92.* OECD Publishing, Paris. http://www.oecd.org/officialdocuments/publicdisplaydocumentpdf/?cote=env/jm/%20mono(2020)32&doclanguage=en

OECD (2020c). Advancing Adverse Outcome Pathway (AOP) Development for Nanomaterial Risk Assessment and Categorisation Part 1: Final Project Report and Recommendations with Methodology to Prioritise Key Events (KEs) Relevant for Manufactured Nanomaterials. *OECD Series on the Safety of Manufactured Nanomaterials No. 93.* OECD Publishing, Paris.

http://www.oecd.org/officialdocuments/publicdisplaydocumentpdf/?cote=env/jm/%20mono(2020)33&doclanguage=en
OECD (2020d). Advancing Adverse Outcome Pathway (AOP) Development for Nanomaterial Risk Assessment and Categorisation Part 2: Case Study on Tissue Injury. *OECD Series on the Safety of Manufactured Nanomaterials No. 94.* OECD Publishing, Paris. https://www.oecd.org/officialdocuments/publicdisplaydocumentpdf/?cote=env/jm/%20mono(2020)34&doclanguage=en
OECD (2020e). Advancing Adverse Outcome Pathway (AOP) Development for Nanomaterial Risk Assessment and Categorisation Part 3: Workshop Report and Recommendations. *OECD Series on the Safety of Manufactured Nanomaterials No. 95.* OECD Publishing, Paris. https://www.oecd.org/officialdocuments/publicdisplaydocumentpdf/?cote=env/jm/%20mono(2020)35&doclanguage=en
